# Is muscle stiffness a determinant for range of motion in the leg muscles?

**DOI:** 10.5114/biolsport.2024.131821

**Published:** 2023-06-10

**Authors:** Marina M. Reiner, Markus Tilp, Masatoshi Nakamura, Andreas Konrad

**Affiliations:** 1 Institute of Human Movement Science, Sport and Health, Graz University, Graz, Austria; 2 Faculty of Rehabilitation Sciences, Nishi Kyushu University, Ozaki, Kanzaki, Saga, Japan

**Keywords:** Muscle stiffness, Hamstrings, Triceps surae, Rectus femoris, Range of motion, Flexibility

## Abstract

Previous training studies with comprehensive stretching durations have reported that an increase in range of motion (ROM) can be related to decreases in muscle stiffness. Therefore, the purpose of this study was to analyze the association between the passive muscle stiffness of three muscle groups (triceps surae, quadriceps, hamstrings) to the respective joint ROM. Thirty-six healthy male soccer players volunteered in this study. After a standardized warm-up, the muscle stiffness was tested via shear wave elastography in six muscles (gastrocnemius medialis and lateralis, rectus femoris, semitendinosus, semimembranosus, and biceps femoris long head). The hip extension, hip flexion, and ankle dorsiflexion ROM were also assessed with a modified Thomas test, a sit and reach test, and a standing wall push test, respectively. We found significant moderate to large correlations between hip flexion ROM and muscle stiffness for the semimembranosus (rP = –0.43; P = 0.01), biceps femoris long head (rP = –0.45; P = 0.01), and overall hamstring stiffness (rP = –0.50; P < 0.01). No significant correlations were found for triceps surae (rP = –0.12; P = 0.51 to 0.67) and rectus femoris muscle stiffness (rP = 0.25; P = 0.14) with ankle dorsiflexion and hip extension ROM, respectively. We conclude that muscle stiffness is an important contributor to hip flexion ROM, but less important for hip extension or ankle joint ROM. Additional contributors to ROM might be tendon stiffness or stretch/pain tolerance.

## INTRODUCTION

Methods such as stretching and foam rolling can increase the range of motion (ROM) acutely (stretching [1–3], foam rolling [4, 5], or the combination of stretching and foam rolling [6, 7]]) as well as in the long term (stretching [8, 9], foam rolling [10]). Two major mechanisms have been reported to be involved in the changes in ROM. On the one hand, an increase in stretch tolerance (i.e., higher tolerated torque) seems to be the most common mechanism for ROM increases after both an acute stretch or foam rolling intervention [11, 12] and after long-term interventions with these modalities [13, 14]. On the other hand, a decrease in muscle stiffness has been reported to be another mechanism for an increase in ROM after acute static and proprioceptive neuromuscular facilitation stretching (but not after dynamic stretching) interventions of > 60 s, as well as after foam rolling [15, 16]. Such decreases in muscle stiffness have also been observed following several weeks of highvolume stretching (i.e., > 30 min a week per muscle group) [17], but not following long-term foam rolling [13]. These training-induced changes in muscle stiffness after stretching (acute and long term) and foam rolling (acute) indicate a causal correlation between changes in muscle stiffness and ROM. However, to date, it is not clear if joint ROM is related to stiffness of the surrounding muscle groups. While studies have reported a correlation between some leg muscle stiffness and ROM, this seems to depend on age, sex, and muscle [18–20]. More specifically, Hirata et al. [20] reported a significant correlation between gastrocnemius medialis and gastrocnemius lateralis muscle stiffness and ankle dorsiflexion ROM in young but not in older participants, measured in a 15° dorsiflexion position. In addition, Miyamoto et al. [18] reported such correlations at 0° ankle angle (gastrocnemius medialis + gastrocnemius lateralis to ankle dorsiflexion ROM) in young male participants but not in young female participants. Moreover, a correlation between the hamstring muscles (semimembranosus, semitendinosus, and biceps femoris long head) and hip flexion ROM was detected in young participants, but without analyzing sex-specific relationships [19]. This was in line with an earlier study which reported that hip flexion ROM is limited by hamstring muscle-tendon unit stiffness [21], without distinguishing between isolated muscle and tendon stiffness, respectively. However, to the best of our knowledge, no study to date has analyzed the association between rectus femoris muscle stiffness and hip extension ROM. Furthermore, no study to date has performed a correlation analysis of all joints and related muscles in the leg within one project.

Additionally, concerning soccer players it is well known that lower joint ROM [22] and higher muscle stiffness [23] can lead to a higher injury prevalence. Thus, it would be important to understand the association between lower leg ROM to muscle stiffness especially in soccer players.

Therefore, the aim of this study was to investigate the correlations between the passive muscle stiffness of three muscle groups (triceps surae, quadriceps, and hamstrings) and the respective joint ROM in recreational soccer players. We hypothesized that local muscle stiffness would correlate with the respective joint ROM.

## MATERIALS AND METHODS

### Participants

An a priori power analysis, based on the results of Hirata et al. [20] revealed an optimal sample size of 27 participants (correlation: bivariate normal model, pH1 = 0.495, α = 0.05, β = 0.80). Therefore, to account for dropout, we recruited 36 healthy male, recreational soccer players from 3^rd^ to 6^th^ Austrian league (training frequency: 3 to 4 times per week + 1 game at the weekend, age: 23.36 ± 4.11 years; height: 181.8 ± 5.2 cm; body mass: 81.2 ± 6.8 kg) to participate in this study. Minimum 6 month prior the study participants were free of any injuries or neuromuscular disorders. The participants were asked to avoid strenuous exercises 72 h prior to the test and should avoid physical training on the test day before the test. All participants signed a written informed consent form. The study was approved by the Ethics Committee of the University of Graz (approval code: GZ. 39/68/63 ex 2020/21) and was performed according to the Declaration of Helsinki.

### Experimental design

Participants visited the laboratory on two separate days. The first appointment was to familiarize the participants with the test procedure. During the second appointment, the data acquisition in the dominant leg (used for kicking a ball) was undertaken. Prior to the measurements, each participant performed a 5-min warm-up on a stationary cycle ergometer (Monark, Ergomedic 874 E, Sweden) at a cadence of 60 rev/min [24] and a resistance of 60 W. Following the warm-up and after positioning the participant for the measurement (about 5 min in between warm up and start of the first measurement) shear wave elastography (SWE) of the dominant leg of six leg muscles (gastrocnemius medialis and gastrocnemius lateralis, rectus femoris, semitendinosus, semimembranosus, and biceps femoris long head) was performed to determine muscle shear modulus as an indicator for muscle stiffness. The ROM of ankle dorsiflexion (standing wall push), hip extension (modified Thomas test), and hip flexion (sit and reach test) was then tested. During the SWE measurements, the surface electromyography (sEMG) was visually monitored on one muscle of each of the three muscle groups of the leg (gastrocnemius lateralis, vastus lateralis, and biceps femoris long head), which allowed us to confirm that the participant was in a rested state.

### Measurements

#### Shear wave elastography (SWE)

The SWE values were measured with an ultrasound scanner (Aixplorer V12.3, Supersonic Imaging, Aix-en-Provence, France) in combination with a linear transducer array (4–15 MHz, SuperLinear 10-2, Vermon, Tours, France) in the six leg muscles. The measurements were done by a qualified tester with ~4 years experience who tested all subjects. The scanner was used in SWE mode (musculoskeletal preset, penetration mode, smoothing level 5, persistence off, scale 0–300 kPa). Per muscle, 3 videos with 15 s each were obtained. The SWE values were analyzed with MATLAB R2017b (Math-Works, Natick USA) and the mean of five consecutive frames with the lowest SD within the range of interest within each video was calculated [25]. The final values for the muscle stiffness were calculated as the mean between the two closest values of the three videos and was divided by 3 to convert the shear wave speed to shear modulus [25]. A handheld technique without any stabilizing support or guiding rail was utilized during the measurements [26, 27]. The tester needed to keep the same probe position without any movement during the whole measurement duration.

To measure the shear modulus of the plantar flexor muscles, the participant was positioned prone in a dynamometer (CON-TREX MJ, CMV AG, Duebendorf, Switzerland) with the hip and knees fully extended (180°, respectively) and the ankle at neutral position (90°). The GM was first measured around the proximal third between the calcaneus and the popliteal fossa. The gastrocnemius lateralis was then measured at the same distance between the heel and knee but on the lateral side of the calf. For the SWE measurements in the rectus femoris, the participant was seated on a dynamometer, while the knee angle was set to 70° and the hip remained at 110° [28]. The rectus femoris was measured around the distal third of the distance between the proximal edge of the patella and the iliac spine [29]. For the shear modulus measurement of the hamstring muscles, the participant was positioned next to the dynamometer in a supine position with a hip angle of 90° and knee angle of 120° to achieve a slightly stretched position of the hamstring muscles [28]. The measuring position for the semitendinosus was distal to the tendinous insertion [25, 30] and the measurement of the biceps femoris long head was performed about half way between the popliteal fossa and the ischial tuberosity on the lateral side of the back thigh [25, 30]. The semimembranosus was measured more medial and more distal than the measuring position of the semitendinosus [25, 30].

The measurement position of the transducer for each muscle was determined during the familiarization session and was marked on a reusable foil [16]. The probe was aligned with fascicle orientation and kept in place for the whole measurement process [31]. Pressure on the skin was avoided to not influence tissue or muscle structure [32]. A conditioning procedure with passive stretches controlled in the dynamometer was performed prior to the SWE to guarantee the same muscle condition in all participants. The angle range of the conditioning was the same for all participants and was chosen carefully to not stretch the tissue too much prior the SWE measurement. The range of interest (ROI) during the measuring process was set centrally and maximized as much as possible, but without including any aponeuroses. The participant was asked to relax completely and avoid any movement during the measurement. This was confirmed by sEMG, as values up to 5% of maximal isometric contraction activation were tolerated. For each muscle, three videos of 15 s each were recorded. For the analysis, the mean of five consecutive frames with the lowest standard deviation of the averaged shear modulus of the ROI within each video was considered. To calculate the mean passive stiffness of a muscle, the two closest mean values out of the three videos were taken [25]. The reliability of all the SWE assessments in any muscle was confirmed in previous experiments [4, 16, 33]. Furthermore, the mean SWE values of all the respective muscle groups where more than one muscle was assessed (i.e., plantar flexors (gastrocnemius medialis + gastrocnemius lateralis), hamstrings (semitendinosus + semimembranosus + biceps femoris long head)) were also calculated as a proxy for overall muscle group stiffness.

### Range of motion (ROM)

The ROM measurements of the dorsiflexion and hip extension were tracked with a 3D motion capture system (Qualisys, Gothenburg, Sweden). Eight cameras were used, and reflective markers (diameter: 1 cm) were positioned on the participant’s hip and dominant leg according to the Qualisys Gait module “CAST lower body marker set”. Two additional markers were positioned on the right and left iliac crest to ensure proper tracking during the hip extension ROM in supine position. Firstly, the dorsiflexion ROM was tested with the standing wall push exercise. The exercise was repeated three times for 5 s each time. The starting position was standing upright in front of a wall. The hands were positioned on the wall at shoulder height and width. After the start command, the participant was asked to move the dominant leg behind the body as far as possible and position it with extended leg and the heel touching the ground. The toes of both legs were front facing. To reach the maximum dorsiflexion ROM at the point of discomfort in the stretched calf muscles of the dominant leg, the knee of the other leg could also be flexed. To test hip extension ROM, the participant was asked to perform three modified Thomas tests [34] with the dominant leg, each for 5 s (in the end position). In each test, the participant lay supine on a medical bed, with the gluteal fold right behind the edge of the bed, and the hip was flexed to 90° with knees fixed by hands with extended elbow joints. The extended elbows ensured the same positioning for each participant and also helped to maintain the contact of the lumbar spine with the medical bed during the test to avoid pelvic tilt during the movement [35]. The participant was asked to relax their legs completely. The contralateral leg was held in position with both hands while the dominant leg was lowered unassisted toward the floor until the end position in a relaxed state was reached. Moreover, to test hip flexion ROM, the participant performed three sit and reach tests with the help of a Sit n’ Reach trunk flexibility box (Fabrication Enterprises; Baseline Model 12-1086, New York, USA). The participant was positioned sitting on the ground in front of the flexibility box with the whole sole of each foot touching the box and the knees fully extended and relaxed. For the starting position, the trunk was kept upright and the arms were held parallel to the ground. The task was to move the slider on top of the flexibility box slowly as far in the direction of the toes (and further) as possible. The knees were kept in a completely extended position during the forward bend procedure. Moreover, both hands were on top of each other during the pushing phase to minimize possible trunk rotation during the hip flexion. The value reached in the maximum forward bend position was noted.

The camera system was calibrated at the beginning of each test day and the data of each trial was controlled for completeness after the measurement. Only trials with clear visibility of all markers during the ROM movement were taken for analysis. If the data of a trial was not complete (i.e., markers were missing) one more trial was conducted. For the analyzing procedure, the data points of the reflective markers were labeled within Qualisys and then exported to Visual 3D, a biomechanical modeling software (Velamed GmbH – Science in Motion, Köln, Germany) to calculate the joint angles within the single ROM-tests. These joint angles were exported to a spreadsheet and the best attempt out of the three was then chosen for further analysis. If an evasive movement in any joint in any of the tests was detected, the attempt was repeated.

### Surface electromyography (sEMG)

SEMG (Myon320, myon AG, Zurich, Switzerland) was used to monitor the muscle activation during SWE testing. Skin preparation and surface electrode positioning (BlueSensor N, Ambu, A/S, Ballerup, Denmark) were performed according to SENIAM recommendations [36] on the muscle belly of the vastus lateralis, biceps femoris long head, and gastrocnemius lateralis. The signal was sampled at 2000 Hz and normalized by a maximal voluntary isometric contraction. If any muscle activation was detected during the SWE assessments (exceeding 5% of maximal muscle contraction, [37]), the trial was repeated. The data were checked live during the SWE assessment. If any abnormality was found during the SWE assessment in the raw sEMG the data was further processed by performing a high-pass filtered (10 Hz Butterworth) and root-mean square (RMS, 50 ms window).

### Statistics

For the statistical analysis, SPSS (version 28, SPSS Inc., Chicago, Illinois) was used and the normal distribution was tested with the Kolmogorov-Smirnov test. In the case of a normal distribution, Person’s correlation coefficient (rP) was used to determine the correlations between the ROM and SWE variables of the respective joints. If the values showed no normal distribution (semitendinosus shear modulus data only), Spearman’s rho (rS) was calculated. The effect size of the correlation coefficients was assessed according to the suggestions of Hopkins [38], i.e., trivial (0–0.1), small (0.1–0.3), moderate (0.3–0.5), large (0.5–0.7), very large (0.7–0.9), and nearly perfect or perfect (0.9–1). The 95% confidence intervals (CIs) for the correlations were also calculated. The alpha level was set to 0.05.

## RESULTS

### Correlation analysis of plantar flexor muscle shear modulus (i.e., stiffness) and ankle dorsiflexion range of motion

The correlation analysis revealed no significant relationship between the muscle shear modulus of the gastrocnemius medialis (rP = –0.12; P = 0.51; 95% CI = –0.43 to 0.22) or gastrocnemius lateralis (rP = –0.07; P = 0.67; 95% CI = –0.39 to 0.26) and the dorsiflexion RoM.

Moreover, no correlation was detected between the mean shear modulus of the gastrocnemii (gastrocnemius medialis +gastrocnemius lateralis) and the dorsiflexion ROM (rP = –0.12; P = 0.51; 95% CI = –0.43 to 0.22).

### Correlation analysis of rectus femoris muscle shear modulus (i.e., stiffness) and hip extension range of motion

The correlation analysis revealed no significant relationship between the muscle shear modulus of the rectus femoris (rP = 0.25; P = 0.14; 95% CI = –0.09 to 0.53) and the hip extension ROM.

### Correlation analysis of hamstring muscle shear modulus (i.e., stiffness) and hip flexion range of motion

The correlation analysis revealed a significant moderate negative relationship between the shear modulus of the semimembranosus (rP = –0.43; P = 0.01; 95% CI = –0.67 to –0.12) and biceps femoris long head (rP = –0.45; P = 0.01; 95% CI = –0.68 to –0.14) and hip flexion ROM. However, there was no correlation between the semitendinosus (rS = –0.10; P = 0.57; 95% CI = –0.42 to 0.25) and hip flexion ROM.

Moreover, a significant large correlation was detected between the mean shear modulus of the hamstring muscles (semimembranosus + semitendinosus + biceps femoris long head) and the hip flexion ROM (rP = 0.50; P < 0.01; 95% CI = –0.71 to –0.21).

**FIG. 1:**
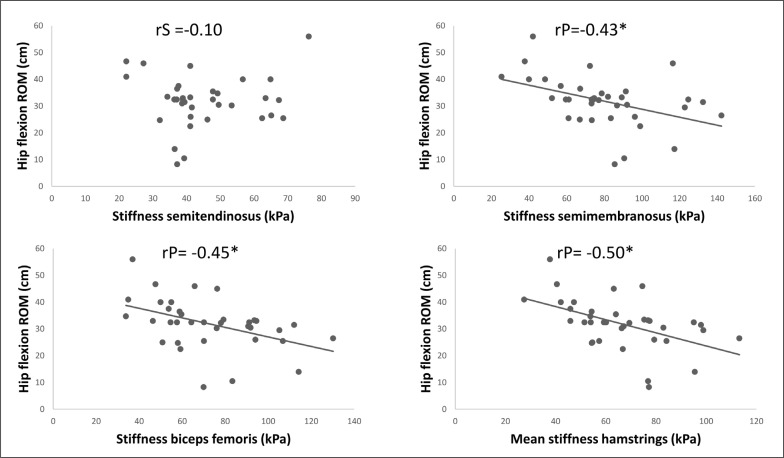
Scatter plots of the correlation between hamstring muscles stiffness assessed with shear wave elastography and hip flexion range of motion. * indicates a significant correlation.

The scatter plots for all the correlations of the hamstring muscle SWE to hip flexion ROM are presented in Figure 1.

## DISCUSSION

The purpose of this study was to investigate if passive muscle stiffness of three muscle groups (triceps surae, quadriceps, and hamstrings) is related to the respective joint ROM. We found a significant small to large negative correlation between hip flexion ROM and the stiffness of the semimembranosus (rP = –0.43), biceps femoris long head (rP = –0.45), and the overall hamstrings (rP = –0.50), which indicates that higher stiffness causes lower hip flexion ROM.

However, in the third hamstring muscle, the semitendinosus, we did not find such a correlation. Moreover, there was no significant correlation between ankle dorsiflexion ROM and gastrocnemius medialis or gastrocnemius lateralis stiffness. Similarly, there was no significant correlation between rectus femoris stiffness and hip extension ROM.

Since previous studies have reported a significant correlation between gastrocnemius medialis and gastrocnemius lateralis stiffness assessed via SWE and dorsiflexion ankle ROM in young men [18, 20], it was surprising that we could not confirm this result in the present study. Differences cannot be explained by the participants as all the studies included young males of a similar age (Hirata et al. [20] age = 22; Miyamoto et al. [18] age = 21.6; current study age = 23). However, the previous studies did not specify whether their participants were athletes or not. We recruited recreational soccer players of the 3^rd^ to 6^th^ Austrian league, and hence it can be assumed that, besides age [20] and sex [18], the training status of the participants may have also affected the correlation between gastrocnemius medialis or gastrocnemius lateralis stiffness and ankle dorsiflexion ROM. This was confirmed in previous studies which reported lower muscle stiffness in untrained participants compared to their athlete peers [39]. Although the muscle stiffness might be higher in athletes, a recent meta-analysis showed that regular strength training can increase the ROM of a joint [40]. Consequently, mechanisms other than muscle stiffness, such as stretch tolerance, may be responsible for the relatively high ankle dorsiflexion ROM found in our sample (36.99 ± 5.37) [41]. Another explanation for the difference in results may be the assessment of the gastrocnemius medialis and gastrocnemius lateralis stiffness, which was performed in a neutral ankle joint position in the present study. Miyamoto et al. [18] and Hirata et al. [20] assessed gastrocnemius medialis and gastrocnemius lateralis stiffness in a slightly stretched position (Miyamoto et al. [18] 14° ankle angle; Hirata et al. [20] 15° ankle angle). When Miyamoto et al. [18] assessed stiffness at a neutral position, the significant correlation with ROM only remained in the gastrocnemius lateralis but not in the GM. No significant correlation was observed below slack length. Consequently, it is likely that such a correlation is dependent on the muscle-tendon unit length.

For the hamstrings, we found significant correlation between hip flexion ROM and the stiffness of the semimembranosus (rP = –0.43), biceps femoris long head (rP = –0.45), and overall hamstrings (rP = –0.50), but not for the semitendinosus (rS = –0.10). In contrast, Miyamoto et al. [19] found a significant correlation between the sit and reach score and all three tested hamstring muscles (semimembranosus (rP = –0.25), biceps femoris long head (rP = –0.263), semitendinosus (rP = –0.299)) and overall hamstring stiffness (rP = –0.331). The slightly less pronounced correlation compared to the present study could be explained by the female participants included in the study by Miyamoto et al. [19]. Previous studies have reported that young males, but not females, showed a significant correlation between gastrocnemius medialis and gastrocnemius lateralis stiffness to ankle dorsiflexion [19]. Consequently, it can be speculated that the correlation between hamstring stiffness and hip flexion ROM might also be sex-dependent. However, Miyamoto et al. [19] did not distinguish between sex in their study, so this remains an open question Another possible explanation for the more pronounced correlations compared to Miyamoto et al. [19] could be that the participants in the current study were recreational male soccer players. All in all, the correlations found in the study of Miyamoto et al. [19] and in the current study range from 0.25 to 0.5, and hence the effect sizes can be considered as small to large. Thus, only 6% to 25% of the variation in ROM can be explained by the variation in muscle stiffness, according to these findings. Consequently, the remaining variation might be explained by other mechanisms, such as stretch tolerance, tendon stiffness, or nerve stiffness.

To the best of our knowledge, this study was the first to explore the correlation between rectus femoris stiffness and hip extension ROM. However, no significant correlation was found between those two variables. Although previous studies have found an increase in ROM following a single bout of foam rolling, no changes in rectus femoris elongation (i.e., indication for stiffness) were reported [42]. Consequently, due to this lack of correlation found by Vigotsky et al. [42], as well as the lack of correlations found in this study, other structures such as the iliopsoas muscle, ligaments, or the joint capsule rather than the rectus femoris muscle could likely explain hip extension ROM.

This study does have some limitations. Firstly, we did not assess tendon stiffness. Since it is not recommended to assess tendon stiffness with SWE, due to the technical restrictions of the device [43], this parameter was not included. It is likely that Achilles tendon stiffness and patellar tendon stiffness might be related to ankle dorsiflexion and hip extension ROM, respectively. Consequently, future studies should aim to assess tendon stiffness via force-elongation curves [24] or other reliable methods such as the use of a Myoton-Pro device [44]. Additionally, through pilot studies we recognized that it was not possible to assess muscle stiffness with SWE of deep lying muscles such as the iliopsoas as well as the soleus muscle with high reliability. Consequently, we decided not to include these muscles into that study. Furthermore, we did not assess stretch tolerance, which is another likely candidate for a correlation with ROM [21]. Finally, we did not include female participants. Since there have been differences reported in the correlation between ROM and muscle stiffness between males and females in a non-athlete population [18], future studies should take this into account.

## CONCLUSIONS

It can be concluded that a small to large correlation exists between hip flexion ROM and the stiffness of the semimembranosus, biceps femoris long head, and overall hamstrings (but not in the semitendinosus). However, it has to be noted that a maximum of 25% of the variation in hip flexion ROM can be explained by muscle stiffness. Moreover, we did not find a significant correlation between ankle dorsiflexion ROM and gastrocnemius medialis or gastrocnemius lateralis stiffness. In addition, there was no significant correlation between rectus femoris stiffness and hip extension ROM. Consequently, other structures such as tendon stiffness or stretch tolerance might be factors which can be related to ankle dorsiflexion ROM and hip extension ROM.
